# The role of the immunoescape in colorectal cancer liver metastasis

**DOI:** 10.1371/journal.pone.0259940

**Published:** 2021-11-19

**Authors:** Chie Takasu, Shoko Yamashita, Yuji Morine, Kozo Yoshikawa, Takuya Tokunaga, Masaaki Nishi, Hideya Kashihara, Toshiaki Yoshimoto, Mitsuo Shimada

**Affiliations:** Department of Surgery, Tokushima University, Tokushima, Japan; VA Boston Healthcare System, Harvard Medical School (Brigham and Women’s Hospital), UNITED STATES

## Abstract

The expression of programmed death 1 (PD-1) and programmed death-ligand 1 (PD-L1) indicate the efficacy of anti-PD-1/PD-L1 therapy in colorectal cancer (CRC), but are less useful for monitoring the efficacy of therapy of CRC liver metastasis (CRLM). This study investigated the effects of immune molecules on the prognosis of CRLM. We enrolled 71 patients with CRLM who underwent curative resection for CRC. We used immunohistochemistry to analyze the expression of PD-1, PD-L1, indoleamine-pyrrole 2,3-dioxygenase (IDO), and CD163 (a marker of tumor-associated macrophages [TAMs]) in metastatic tumors. The immune molecules PD-1, PD-L1, IDO, and TAMs were expressed in 32.3%, 47.8%, 45.0%, and 47.9% of metastatic CRC samples, respectively. The 5-year overall survival rates associated with immune molecule-positive groups were significantly better than in the negative groups (PD-1: 87.7% vs 53.2%, *p* = 0.023; PD-L1: 82.4% vs 42.3%, *p* = 0.007; IDO: 80.7% vs 43.5%, *p* = 0.007; TAMs: 82.6% vs 48.0%, *p* = 0.005). Multivariate analysis revealed PD-1 expression (*p* = 0.032, hazard ratio: 0.19), IDO expression (*p* = 0.049, hazard ratio: 0.37), and tumor differentiation (*p<*0.001, hazard ratio: 0.02) as independent prognostic indicators. PD-1 and TAMs in metastases were associated with less aggressive features such as smaller tumors. Furthermore, TAMs positively and significantly correlated with PD-1 expression (*p* = 0.011), PD-L1 expression (*p* = 0.024), and tended to correlate with IDO expression (*p* = 0.078). PD-1, PD-L1, IDO, and TAMs in CRLM were associated with less aggressive features and better prognosis of patients with CRC, indicating adaptive antitumor immunity vs immune tolerance. These molecules may therefore serve as prognostic markers for CRLM.

## Introduction

Colorectal cancer (CRC) is the third most common malignancy worldwide and was the second leading cause of cancer death in 2018 [1]. The incidence of CRC, which is steadily increasing, widely varies among countries [2]. Approximately 25% of patients with CRC present with liver metastasis upon diagnosis, and approximately 50% develop liver metastasis over the course of their disease [3]. Therefore, control of CRC liver metastasis (CRLM) is critical for improving prognosis.

Multiple reports demonstrate a link between metastasis and the immune system. For example, several types of immune cells mediate the inhibition and promotion of metastatic disease [4, 5]. Furthermore, the immune response to a progressive primary tumor inhibits metastasis, which involves the activity of suppressor cells and the secretion of inhibitory factors by the primary tumor. This activity is lost after primary tumor resection, leading to the progression of metastasis [6]. In contrast, other reports show that the primary tumor induces a persistent immune response to the metastatic site even after primary tumor resection [7].

Metastatic tumors do not initially occupy an immune-suppressive environment and defense system against the host immune system as observed in primary tumors. Therefore, metastatic tumors may be easily detected and killed by the immune response. Numerous tumor-infiltrating lymphocytes (TILs) reside in metastatic sites [8], suggesting that the host immune response protects against the progression of metastatic disease. These findings indicate that the immune response against metastatic tumors differs from that targeting primary tumors.

Recent research on the tumor microenvironment focuses on immune cells as well as the immunoescape system, including immune checkpoint molecules. We previously reported that the expression of immune molecules such as programmed death 1 (PD-1), programmed death-ligand 1 (PD-L1), and indoleamine-pyrrole 2,3-dioxygenase (IDO) is associated with poor prognosis and immunotolerance through induction of the activation of regulatory T cell (Treg cell) in gastric cancer and CRC [9–11]. PD-L1 expression in tumor cells predicts the response to an anti-PD-1 blockade [12]. Furthermore, PD-L1 expression is higher in lung [8] and liver [13] metastases of patients with CRC compared with the cognate primary tumors. If immunity functions are similar in the metastatic lesions and primary tumors, the efficacy of PD-1 blockade against these metastatic lesions may predict a good response to treatment. Moreover, immune-related therapies are more likely applied to CRLM than resectable CRC. Therefore, the expression of immune molecules in metastatic CRC may identify patients who may benefit from immune-related therapy.

We previously reported that cell migration and the epithelial–mesenchymal transition is stimulated by tumor-associated macrophages (TAMs) in hepatocellular carcinoma [14]. Liver macrophages are required for the immune response to cancer cells [15]. Macrophages are activated in the tumor microenvironment by cytokines to undergo specific polarization. The relevant macrophage phenotypes include antitumor, classically activated M1 macrophages, and the protumor alternatively activated M2 macrophages. Tumor-associated macrophages (TAMs) exhibit a predominantly M2-like phenotype because they promote angiogenesis and invasion [16].

However, the prognostic significance of immune molecules in CRLM is controversial and incompletely understood. Therefore, here we used immunohistochemistry to evaluate the expression of PD-1, PD-L1, and IDO as well as the frequency of TAMs to determine their functions and prognostic significance in CRLM.

## Patients and methods

### Patients

We enrolled 71 patients with CRLM who underwent curative (R0) hepatic resection at Tokushima University Hospital from 1995 to 2013. The mean follow-up was 51.9 months (range: 4–185 months). We excluded patients who underwent noncurative surgery (R1 or R2) for primary tumors or CRLM or those who had remote organ metastasis. Liver metastasis, H-stage classification, and grade classification were defined according to the Japanese Classification of Colorectal Carcinoma, Second English Edition [17]. H-stage was determined according to the number and largest size of liver metastasis as follows: H1, <4 metastases, diameter ≤5 cm; H2, >5 metastases or diameter >5 cm; and H3, >5 metastases, diameter >5 cm. Grade classifications, which included H-stage, mesenteric lymph node metastases (pN0/1: ≤3 lesions, pN2: ≥4 lesions), and extrahepatic metastases (EM0: absence of extrahepatic metastasis, EM1: presence of extrahepatic metastases), were as follows: Grade A: H1 and pN0/1; Grade B: H2 and pN0/1 or H1 and pN2; and Grade C: H3, any pN, any H-stage, and any pN with EM1.

A nomogram published by the Japanese Society of Hepato-Biliary-Pancreatic Surgery served as a prognostic scoring system after hepatic resection for CRLM [18]. Each participant provided written informed consent, and the Institutional Review Board of the University of Tokushima Graduate School authorized this study in advance (No. 2395 authorized in 2015).

### Immunohistochemistry

The Department of Pathology, Tokushima University Hospital routinely performs hematoxylin and eosin staining to diagnose cancer. Hematoxylin and eosin-stained sections were used to confirm the location of a cancer.

Tissue samples for immunohistochemistry were fixed in formalin and embedded in paraffin. Samples were cut into 5-μm-thick serial sections, which were dewaxed, deparaffinized in xylene, and rehydrated using a series of decreasing alcohol concentrations. Samples were boiled for 20 min in a microwave oven in citrate buffer (pH 6.0) for antigen retrieval. The sections were incubated in Protein Block Serum-Free Reagent (DAKO, Carpinteria, USA) for 30 min to block nonspecific binding. The slides were then incubated with primary antibodies overnight at 4°C. The primary antibodies and dilutions were as follows: mouse monoclonal antibody against PD-1 (AF1086, 1:40; R&D Systems, Minneapolis, USA), rabbit monoclonal antibody against PD-L1 (ab174838, 1:100; Abcam, Cambridge, UK), rabbit polyclonal antibody against CD163 as a TAMs marker (ab87099, 1:100; Abcam, Cambridge, UK), and a mouse monoclonal antibody against IDO (ab71276, 1:50; Abcam, Cambridge, UK). Secondary antibody binding to these proteins was detected using a Histofine SAB-PO Kit (Nichirei Biosciences Inc., Tokyo, Japan) for PD-1 and an EnVision Dual Link System-HRP (K4065: Dako Corporation, Carpinteria, USA) for PD-L1, IDO, and TAMs. A secondary peroxidase-labeled polymer conjugated to goat anti-mouse immunoglobulin was applied for 60 min. The sections were developed in 3,3-diaminobenzidine and counterstained with Mayer’s hematoxylin. Each slide was dehydrated using a series of increasing alcohol concentrations and then covered with a coverslip. Sections of human tonsils served as the positive control for PD-1, PD-L1, IDO, and TAMs. The presence of positive cells on each slide was determined by a pathologist uninformed of the origin of the samples. PD-1 and PD-L1 expression was evaluated in the primary tumor as well as the corresponding metastatic tumor.

**Fig 1:**
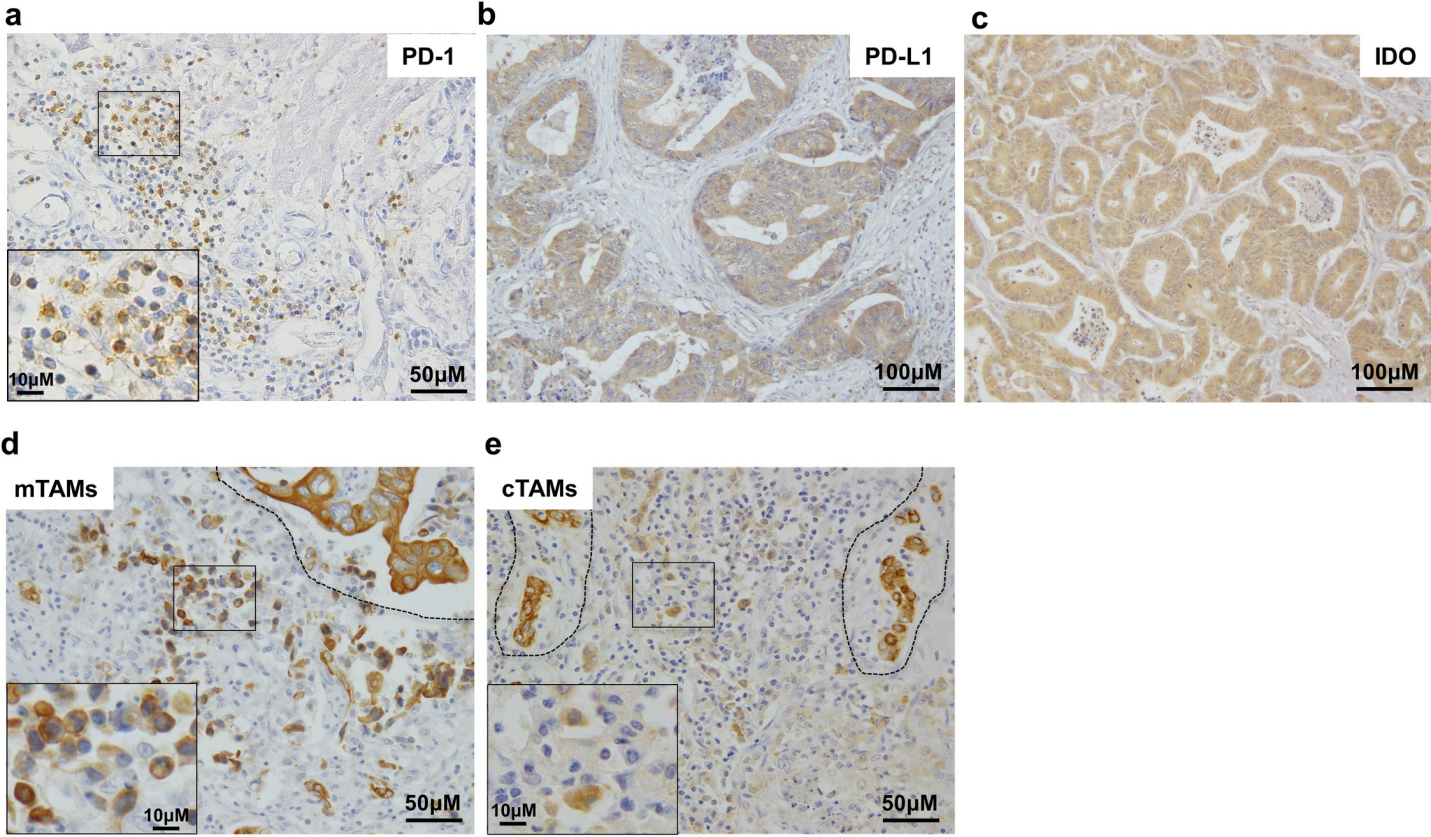
Immunohistochemical analysis of PD-1, PD-L1, IDO, and TAMs expression in colorectal cancer liver metastasis. Representative images are shown. The insets of a, d, and e show high magnification views of the boxed area in each panel. a. PD-1 expression in mononuclear cells in tumor tissue (400×).b. PD-L1 expression in tumor cells (200×). c. IDO expression in tumor cells (200×). d. CD163-positive cells in the marginal areas (mTAMs) of tumor tissues (400×). The dashed line delineates the invasive front with the tumor tissue on the top side and the normal tissue on the bottom side. CD-163-positive cells at the tumor margin were defined as mTAMs. e. CD163-positive cells in the central area (cTAMs) of tumor tissue (400×). The tumor cells are surrounded by the dashed line. CD163-positive cells at the center of tumor tissue were defined as cTAMs.

To evaluate PD-1 status, we examined tumor tissue sections at ×400 magnification using an imaging system (DXM1200F; Melville, USA), and PD-1 expression was recorded when >40% of mononuclear cells in tumor tissues exhibited partial or complete membrane staining by the PD-1 antibody (Fig 1A).

PD-L1 and IDO expression was predominantly cytoplasmic (Fig 1B and 1C), and staining intensity was scored as follows: 0, no staining; 1+, weak staining; 2+, moderate staining; and 3+, strong staining. Distribution scores were determined by calculating the percentage of positive cancer cells and scoring the samples as follows: PD-L1: 0, 0%–5%; 1+, 6%–25%; 2+, 26%–50%; 3+, 51%–75%; and 4+, 76%–100%, IDO: 0, 0%–9%; 1+, 10%–50%; 2+, 51%–80%; 3+, 51%–80%; and 4+, 81%–100%. The final score was calculated as the sum of the staining intensity and distribution scores. A total score >3 was defined as PD-L1-positive expression in tumors [9, 19], and >4 was defined as IDO-positive expression in tumors [20].

To evaluate TAMs, round cells positive for CD163 were identified by screening the entire tumor area at 100× magnification and selecting five areas with the highest density of macrophages. CD163 was strongly stained in tumor cells and the bile duct (Fig 1D and 1E), and we distinguished TAMs from these cells according to their morphologies. The mean percentage of CD163-positive cells counted in five independent 400× fields was calculated, and samples with ≥20% stained cells were defined as high-density TAMs [21, 22]. CD163-positive cells (TAMs) were detected at the tumor margin (mTAMs) (Fig 1D) or at the tumor center (cTAMs) (Fig 1E).

### Statistical analysis

All statistical analyses were performed using JMP 8.0.1 (SAS, Cary, NC, USA). The chi-squared test was used to compare values between two groups according to patients’ clinical information. Continuous variables were compared using the Mann–Whitney test. Survival curves were generated using the Kaplan–Meier method and compared using log-rank tests. Potential prognostic factors were analyzed using univariate analysis. *P* < 0.05 was considered significant.

## Results

### Characteristics of patients and tumors

This study included 71 patients with CRC (48 men and 23 women; mean age 66.9 years, range 32–90 years). The mean follow-up was 51.9 months (range, 4–185 months). The mean interval between primary tumor resection and liver metastasis in metachronous cases was 14.4 months (range, 3.4–74.1 months). Among the 71 patients, 43 had primary colon tumors and 28 had primary rectal tumors. Synchronous and metachronous CRLM was observed in 41 and 30 patients, respectively. Among the 41 patients with synchronous metastasis, one underwent adjuvant chemotherapy after primary tumor resection. Among the 30 patients with metachronous metastasis, 2 underwent adjuvant chemotherapy after primary tumor resection, and 43 (synchronous, 31 patients; metachronous, 12 patients) underwent adjuvant chemotherapy after hepatectomy for CRLM. Some patients underwent treatment at a different hospital before hepatectomy. Therefore, certain preoperative clinical data such as metastatic grade, CEA, CA19-9, and primary tumor features (differentiation, venous invasion, and lymphatic invasion) were not available.

### Correlations between immune molecules and clinicopathological characteristics

**Fig 2:**
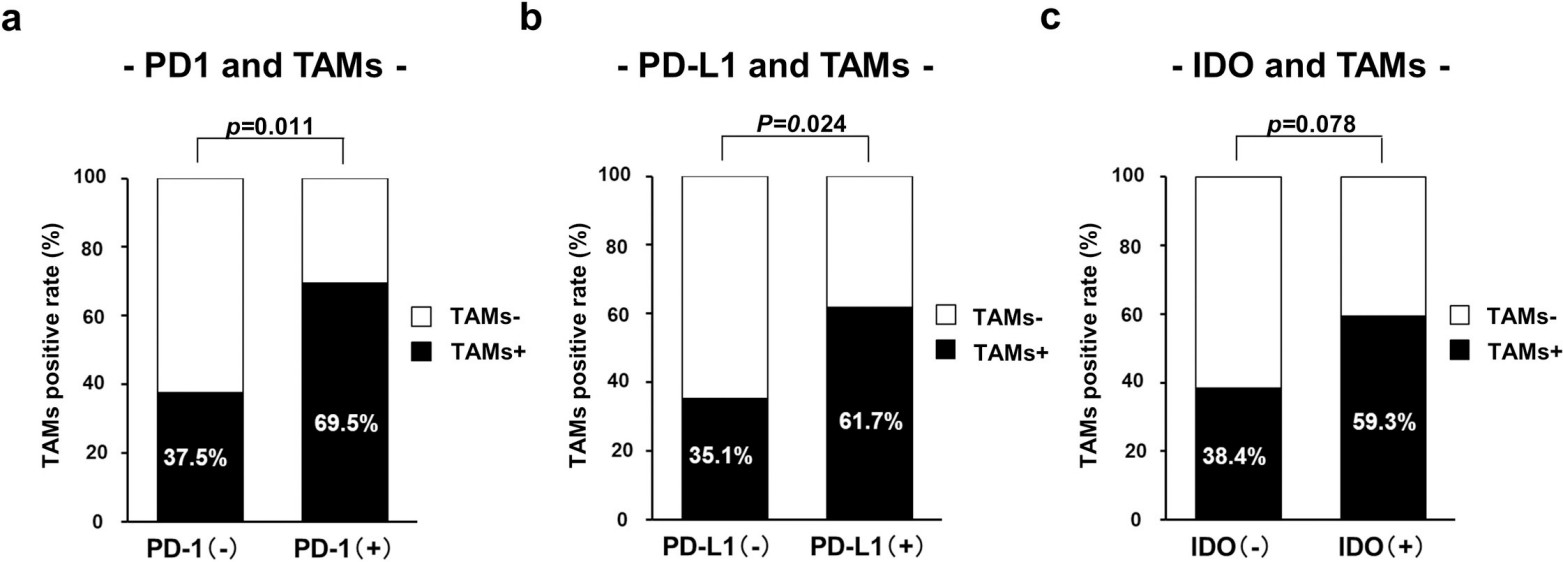
Correlation between TAMs and PD-1, PD-L1, and IDO. Correlation of high density of TAMs with a. PD-1, b. PD-L1, and c. IDO expression in colorectal cancer liver metastasis.

Patients’ clinicopathological characteristics according to PD-1 expression are shown in Table 1. PD-1 expression in metastatic tumors was significantly associated with smaller tumors (*p =* 0.023). PD-1 expression in metastatic tumors significantly correlated with lower (better) nomogram preoperative scores (*p =* 0.006). The rate of administration of postoperative chemotherapy was higher in patients with PD-1-positive metastatic tumors compared with those with PD-1-negative metastatic tumors (*p =* 0.011). PD-1 expression in metastatic tumors was associated with a high density of TAMs (*p =* 0.011) (Fig 2A). Primary tumor characteristics did not significantly differ according to PD-1 expression, and PD-1 expression in metastatic tumors did not significantly correlate with PD-1 and PD-L1 expression in primary tumors (*p* = 0.158 and *p* = 0.824, respectively).

**Table 1:** Patients’ clinicopathological characteristics according to PD-1 expression.

Variables	PD-1 (-)	PD-1 (+)	p-value
	(n = 48)	(n = 23)	
**< Metastatic tumor characteristics >**			
Age (years)	67.1 ± 11.6	66.7 ± 10.7	0.971
Sex (men/women)	32/16	16/7	0.801
	(66.7%/33.3%)	(69.6%/30.4%)	
Tumor maximum size (cm)	4.0 ± 2.2	2.6 ± 0.9	0.023
Tumor number	3.1 ± 2.6	2.3 ± 1.9	0.137
H-stage (H1/H2, 3)	27/21	18/5	0.065
	(56.3%/43.7%)	(78.3%/21.7%)	
Grade (A/B,C)	22/26	16/7	0.058
	(45.8%/55.2%)	(69.6%/30.4%)	
Metastasis period (synch/meta)	31/17	10/13	0.093
	(64.6%/35.4%)	(43.5%/56.5%)	
Pre-operative chemotherapy (-/+)	46/2	23/0	0.207
	(95.8%/4.2%)	(100%/0%)	
Post-operative chemotherapy (-/+)	14/34	14/9	0.011
	(29.2%/70.8%)	(60.9%/39.1%)	
Nomogram preoperative score (<11 / ≥11)	17/31	16/7	0.006
	(35.4%/64.6%)	(69.6%/30.4%)	
CEA (<5 / ≥5) *	20/23	6/16	0.129
	(46.5%/53.5%)	(27.3%/72.7%)	
CA19-9 (<37 / ≥37)*	24/19	17/5	0.083
	(55.8%/44.2%)	(77.3%/22.7%)	
PD-L1 expression (-/+)	28/20	9/14	0.129
	(58.3%/41.7%)	(39.1%/60.9%)	
IDO expression (-/+)	26/22	13/10	0.852
	(54.2%/45.8%)	(56.5%/43.5%)	
TAMs (-/+)	30/18	7/16	0.011
	(62.5%/37.5%)	(30.4%/69.6%)	
**< Primary tumor characteristics >**			
Tumor differentiation (diff./undiff.)	46/2	21/2	0.453
	(95.8%/4.2%)	(91.3%/8.7%)	
T (2,3/4)*	34/12	21/2	0.080
	(73.9%/26.1%)	(91.3%/8.7%)	
Location (colon/rectum)	31/17	12/11	0.319
	(64.6%/35.4%)	(52.2%/47.8%)	
Lymph node metastasis (-/+)	21/27	11/12	0.747
	(43.8%/56.2%)	(47.8%/52.2%)	
Venous invasion (-/+)*	15/31	5/18	0.369
	(32.6%/67.4%)	(21.7%/78.3%)	
Lymphatic invasion (-/+)*	17/28	8/15	0.909
	(37.8%/62.2%)	(34.8%/65.2%)	
PD-1 expression (-/+)	23/25	7/16	0.158
	(47.9%/52.1%)	(30.4%/69.6%)	
PD-L1 expression (-/+)	30/18	15/8	0.824
	(62.5%/37.5%)	(65.2%/34.8%)	

synch/ meta: synchronous/metachronous, diff/undiff.: differentiated histological type/ undifferentiated histological type

*Data for certain patients were unavailable.

Patients’ clinicopathological characteristics according to PD-L1 expression are shown in Table 2. Metastatic tumor characteristics did not significantly differ according to PD-L1 expression. PD-L1 expression in metastatic tumors was significantly associated with IDO expression (*p =* 0.006), a high density of TAMs (*p =* 0.024) (Fig 2B). Regarding tumor characteristics, PD-L1 expression in metastatic tumors was associated with higher differentiation (*p =* 0.020) and fewer lymph node metastasis (PD-L1- vs PD-L1+: 67.6% vs 41.2%, *p =* 0.025). Furthermore, PD-L1 expression in metastatic tumors significantly correlated with PD-L1 expression in primary tumors (*p<*0.001).

**Table 2:** Patients’ clinicopathological characteristics according to PD-L1 expression.

Variables	PD-L1 (-)	PD-L1 (+)	p-value
	(n = 37)	(n = 34)	
**< Metastatic tumor characteristics >**			
Age (years)	68.9 ± 11.5	64.9 ± 11.3	0.287
Sex (men/women)	25/12	23/11	0.994
	(67.6%/32.4%)	(67.6%/32.4%)	
Tumor maximum size (cm)	3.7 ± 2.2	3.3 ± 1.7	0.604
Tumor number	3.0 ± 2.7	2.7 ± 2.1	0.827
H-stage (H1/H2, 3)	23/14	22/12	0.824
	(62.2%/37.8%)	(64.7%/35.3%)	
Grade (A/B,C)	18/19	19/15	0.702
	(48.6%/51.4%)	(55.9%/44.1%)	
Metastasis period (synch/meta)	24/13	17/17	0.205
	(64.9%/35.1%)	(50.0%/50.0%)	
Pre-operative chemotherapy (-/+)	36/1	33/1	0.952
	(97.3%/2.7%)	(97.1%/2.9%)	
Post-operative chemotherapy (-/+)	14/23	14/20	0.774
	(37.8%/62.2%)	(41.2%/58.8%)	
Nomogram preoperative score (<11 / ≥11)	16/21	17/17	0.568
	(43.2%/56.8%)	(50.0%/50.0%)	
CEA (<5 / ≥5) *	16/20	10/19	0.414
	(44.4%/55.6%)	(34.5%/65.5%)	
CA19-9 (<37 / ≥37)*	20/16	21/8	0.158
	(55.6%/44.4%)	(72.4%/27.6%)	
PD-1 expression (-/+)	28/9	20/14	0.129
	(75.7%/24.3%)	(58.8%/41.2%)	
IDO expression (-/+)	26/11	13/21	0.006
	(70.3%/29.7%)	(38.2%/61.8%)	
TAMs (-/+)	24/13	13/21	0.024
	(64.9%/35.1%)	(38.2%/61.8%)	
**< Primary tumor characteristics >**			
Tumor differentiation (diff./undiff.)	33/4	34/0	0.020
	(89.2%/10.8%)	(100%/0%)	
T (2,3/4)*	28/8	28/6	0.632
	(77.8%/22.2%)	(82.4%/17.6%)	
Location (colon/rectum)	24/13	19/15	0.439
	(64.9%/35.1%)	(55.9%/44.1%)	
Lymph node metastasis (-/+)	12/25	20/14	0.025
	(32.4%/67.6%)	(58.8%/41.2%)	
Venous invasion (-/+)*	10/26	10/24	0.880
	(27.8%/72.2%)	(29.4%/70.6%)	
Lymphatic invasion (-/+)*	12/24	13/21	0.669
	(33.3%/66.7%)	(38.2%/61.8%)	
PD-L1 expression (-/+)	34/3	11/23	<0.001
	(91.9%/8.1%)	(32.4%/67.6%)	
PD-1 expression (-/+)	17/20	13/21	0.511
	(45.9%/54.1%)	(38.2%/61.8%)	

synch/meta: synchronous/metachronous, diff./undiff.: differentiated histological type/undifferentiated histological type

*Data for certain patients were unavailable.

Patients’ clinicopathological characteristics according to IDO expression are shown in Table 3. Metastatic tumor characteristics did not significantly differ according to IDO expression. IDO expression in metastatic tumors was significantly associated with PD-L1 expression (p = 0.006). Regarding the primary tumor’s characteristics, IDO expression in metastatic tumors was associated with higher differentiation (*p =* 0.026). Significant differences in other characteristics were not observed.

**Table 3:** Patients’ clinicopathological characteristics according to IDO expression.

Variables	IDO (-)	IDO (+)	p-value
	(n = 39)	(n = 32)	
**< Metastatic tumor characteristics >**			
Age (years)	68.3 ± 12.4	65.37 ± 10.5	0.236
Sex (men/women)	26/13	22 /10	0.852
	(66.7%/33.3%)	(68.8%/31.2%)	
Tumor maximum size (cm)	3.8 ± 2.3	3.1 ± 1.5	0.110
Tumor number	2.9 ± 2.3	2.7 ± 2.6	0.482
H-stage (H1/H2, 3)	22/17	23/9	0.176
	(56.4%/43.6%)	(71.9%/28.1%)	
Grade (A/B,C)	18/21	20/12	0.168
	(46.2%/53.8%)	(62.5%/37.5%)	
Metastasis period (synch/meta)	24/15	17/15	0.475
	(61.5%/38.5%)	(53.1%/46.9%)	
Pre-operative chemotherapy (-/+)	39/0	30/2	0.071
	(100%/0%)	(93.8%/6.2%)	
Post-operative chemotherapy (-/+)	16/23	12/20	0.762
	(41.0%/59.0%)	(37.5%/62.5%)	
Nomogram preoperative score (<11 / ≥11)	19/20	14/18	0.676
	(48.7%/51.3%)	(43.8%/56.2%)	
CEA (<5 / ≥5) *	12/23	14/16	0.310
	(34.3%/65. 7%)	(46.7%/53. 3%)	
CA19-9 (<37 / ≥37)*	23/12	18/12	0.634
	(65.7%/34.3%)	(60.0%/40.0%)	
PD-1 expression (-/+)	26/13	22/10	0.852
	(66.7%/33.3%)	(68.8%/31.2%)	
PD-L1 expression (-/+)	26/13	11/21	0.006
	(66.7%/33.3%)	(34.4%/65.6%)	
TAMs (-/+)	24/15	13/19	0.078
	(61.5%/38.5%)	(40.6%/59.4%)	
**< Primary tumor characteristics >**			
Tumor differentiation (diff./undiff.)	35/4	32/0	0.026
	(89.7%/10.3%)	(100%/0%)	
T (2,3/4)*	30/8	26/6	0.810
	(78.9%/21.1%)	(81.3%/18.7%)	
Location (colon/rectum)	26/13	17/15	0.245
	(66.7%/33.3%)	(53.1%/46.9%)	
Lymph node metastasis (-/+)	16/23	16/16	0.449
	(41.0%/59.0%)	(50.0%/50.0%)	
Venous invasion (-/+)*	12/26	8/24	0.543
	(31.6%/68.4%)	(25.0%/75.0%)	
Lymphatic invasion (-/+)*	11/27	14/18	0.198
	(28.9%/71.1%)	(43.8%/56.2%)	
PD-1 expression (-/+)	18/21	12/20	0.462
	(46.2%/53.8%)	(37.5%/62.5%)	
PD-L1 expression (-/+)	27/12	18/14	0.259
	(69.2%/30.8%)	(56.2%/43.8%)	

synch/meta: synchronous/ metachronous, diff./undiff.: differentiated histological type/undifferentiated histological type

*Data for certain patients were unavailable.

Patients’ clinicopathological characteristics according to TAMs are shown in Table 4. A high density of TAMs in metastatic tumors was significantly associated with less aggressive features such as smaller tumors (*p =* 0.027), lower H stage (*p =* 0.006), lower grade (*p =* 0.021), and lower nomogram preoperative scores (*p =* 0.013). These features mainly reflected the characteristics of mTAMs. A high density of mTAMs in metastatic tumors was significantly associated with smaller tumors (*p =* 0.011), lower H stage (*p =* 0.006), lower grade (*p =* 0.033), and lower nomogram preoperative scores (*p =* 0.027). A high density of TAMs positively and significantly correlated with PD-1 expression (*p =* 0.011) (Fig 2A) and PD-L1 expression (*p =* 0.024) (Fig 2B) in metastatic tumors. There was a positive correlation between TAMs and IDO that was not statistically significant (*p =* 0.078) (Fig 2C). A high density of mTAMs positively and significantly correlated with the expression of PD-1 (*p =* 0.043) and PD-L1 (*p =* 0.046). Regarding to primary tumor characteristics, high densities of TAMs and mTAMs in metastatic tumors were significantly associated with shallow tumor invasion (*p* = 0.019 and *p* = 0.007, respectively).

**Table 4:** Patients’ clinicopathological characteristics according to tumor-associated macrophage (TAMs).

Variables	TAMs		p-value	cTAMs		p-value	mTAMs		p-value
	(-)	(+)		(-)	(+)		(-)	(+)	
	(n = 37)	(n = 34)		(n = 57)	(n = 14)		(n = 40)	(n = 31)	
**< Metastatic tumor characteristics >**									
Age (years)	67.0 ± 12.3	66.9 ± 10.9	0.990	68.0 ± 10.3	62.9 ± 14.0	0.172	66.5 ± 12.2	67.6 ± 10.8	0.719
Sex (men / women)	24/13	24/10	0.606	38/19	10/4	0.731	27/13	21/10	0.983
	(64.9%/35.1%)	(70.6%/29.4%)		(66.7%/33.3%)	(71.4%/28.6%)		(67.5%/22.5%)	(67.8%/32.2%)	
Tumor maximum size (cm)	4.1 ± 2.2	3.0 ± 1.7	0.027	3.5 ± 2.1	3.6 ± 1.9	0.596	4.0 ± 2.1	2.9 ± 1.8	0.011
Tumor number	3.4 ± 2.9	2.3 ± 1.8	0.149	2.9 ± 2.4	2.8 ± 2.7	0.760	3.3 ± 2.8	2.3 ± 1.7	0.181
H-stage (H1/H2, 3)	18/19	27/7	0.006	36/21	9/5	0.937	20/20	25/6	0.006
	(48.6%/51.4%)	(79.4%/20.6%)		(63.2%/36.8%)	(64.3%/35.7%)		(50.0%/50.0%)	(80.6%/19.4%)	
Grade (A/B,C)	15/22	23/11	0.021	29/28	9/5	0.304	17/23	21/10	0.033
	(40.5%/59.5%)	(67.6%/29.4%)		(50.9%/49.1%)	(64.3%/35.7%)		(42.5/57.5%)	(67.7%/32.3%)	
Metastasis period	25/12	16/18	0.080	31/26	10/4	0.239	26/14	15/16	0.160
(synch/meta)	(67.6%/32.4%)	(47.1%/52.9%)		(54.4%/45.6%)	(71.4%/28.6%)		(65.0%/35.0%)	(48.4%/51.6%)	
Pre-operative chemotherapy (-/+)	36/1	33/1	0.952	56/1	13/1	0.330	39/1	30/1	0.855
	(97.3%/2.7%)	(97.1%/2.9%)		(98.2%/1.8%)	(92.9%/7.1%)		(97.5%/2.5%)	(96.8%/3.2%)	
Post-operative chemotherapy (-/+)	11/26	17/17	0.080	23/34	5/9	0.749	13/27	15/16	0.174
	(29.7%/70.3%)	(50.0%/50.0%)		(40.3%/59.7%)	(35.7%/64.3%)		(32.5%/67.5%)	(48.4%/51.6%)	
Nomogram preoperative score	12/25	21/13	0.013	25/32	8/6	0.372	14/26	19/12	0.027
(<11 / ≥11)	(32.4%/67.6%)	(61.8%/38.2%)		(43.9%/56.1%)	(57.1%/42.9%)		(35.0%/65.0%)	(61.3%/38.7%)	
CEA (<5 / ≥5) *	15/19	11/20	0.477	21/32	5/7	0.896	16/20	10/19	0.414
	(44.1%/55.9%)	(35.5%/64.5%)		(39.6%/60.4%)	(41.7%/58.3%)		(44.4%/55.6%)	(34.5%/65.5%)	
CA19-9 (<37 / ≥37)*	21/13	20/11	0.818	33/20	8/4	0.774	22/14	19/11	0.714
	(67.6%/32.4%)	(64.5%/35.5%)		(62.3%/37.7%)	(66.7%/33.3%)		(61.1%/38.9%)	(63.3%/36.7%)	
PD-1 expression (-/+)	30/7	18/16	0.011	39/18	9/5	0.768	31/9	17/14	0.043
	(81.1%/18.9%)	(52.9%/47.1%)		(68.4%/31.6%)	(64.3%/35.7%)		(77.5%/22.5%)	(54.8%/45.2%)	
PD-L1 expression	24/13	13/21	0.024	30/27	7/7	0.859	25/15	12/19	0.046
(-/+)	(64.9%/35.1%)	(38.2%/61.8%)		(52.6%/47.4%)	(50.0%/50.0%)		(62.5%/37.5%)	(38.7%/61.3%)	
IDO expression (-/+)	24/13	15/19	0.078	33/24	6/8	0.312	26/14	13/18	0.052
	(64.9%/35.1%)	(44.1%/55.9%)		(57.9%/42.1%)	(42.9%/57.1%)		(65.0%/35.0%)	(41.9%/58.1%)	
**< Primary tumor characteristics >**									
Tumor differentiation	34/3	33/1	0.334	55/2	12/2	0.160	37/3	30/1	0.425
(diff./ undiff.)	(91.9%/8.1%)	(67.7%/32.3%)		(96.5%/3.5%)	(85.7%/14.3%)		(92.5%/7.5%)	(96.8%/3.2%)	
T (2,3/4) *	25/11	31/3	0.019	45/11	11/3	0.882	27/12	29/2	0.007
	(69.4%/30.6%)	(91.2%/8.8%)		(80.4%/19.6%)	(85.7%/14.3%)		(69.2%/7.8%)	(93.5%/6.5%)	
Location	21/16	22/12	0.493	36/21	7/7	0.371	23/17	20/11	0.548
(colon/rectum)	(56.8%/43.2%)	(64.7%/35.3%)		(63.2%/36.8%)	(50.0%/50.0%)		(57.5%/42.5%)	(64.5%/35.5%)	
Lymph node metastasis	18/19	14/20	0.527	27/30	5/9	0.429	21/19	18/13	0.640
(-/+)	(48.7%/51.3%)	(41.1%/58.9%)		(47.4%/52.6%)	(35.7%/64.3%)		(52.5%/47.5%)	(58.1%/41.9%)	
Venous invasion	10/26	10/24	0.880	16/40	4/10	1.000	11/28	9/22	0.939
(-/+)*	(27.8%/72.2%)	(29.4%/70.6%)		(28.6%/71.4%)	(28.6%/71.4%)		(28.2%/71.8%)	(29.0%/71.0%)	
Lymphatic invasion	13/23	12/22	0.943	21/35	4/10	0.528	13/26	12/19	0.641
(-/+)*	(36.1%/63.9%)	(35.3%/64.7%)		(37.5%/62.5%)	(28.6%/71.4%)		(33.3%/66.7%)	(38.7%/61.3%)	
PD-1 expression (-/+)	19/18	11/23	0.104	28/29	2/12	0.013	20/20	10/21	0.131
	(51.4%/48.6%)	(52.9%/47.1%)		(49.1%/50.9%)	(14.3%/85.7%)		(50.0%/50.0%)	(32.3%/67.7%)	
PD-L1 expression	26/11	19/15	0.208	38/19	7/7	0.253	27/13	18/13	0.414
(-/+)	(70.3%/29.7%)	(55.9%/44.1%)		(66.7%/33.3%)	(50.0%/50.0%)		(67.5%/32.5%)	(58.1%/41.9%)	

synch/meta: synchronous/metachronous, diff./undiff.: differentiated histological type/undifferentiated histological type

* Data for certain patients were unavailable.

### Overall survival (OS) according to immune molecule expression and TAMs in CRLM

**Fig 3:**
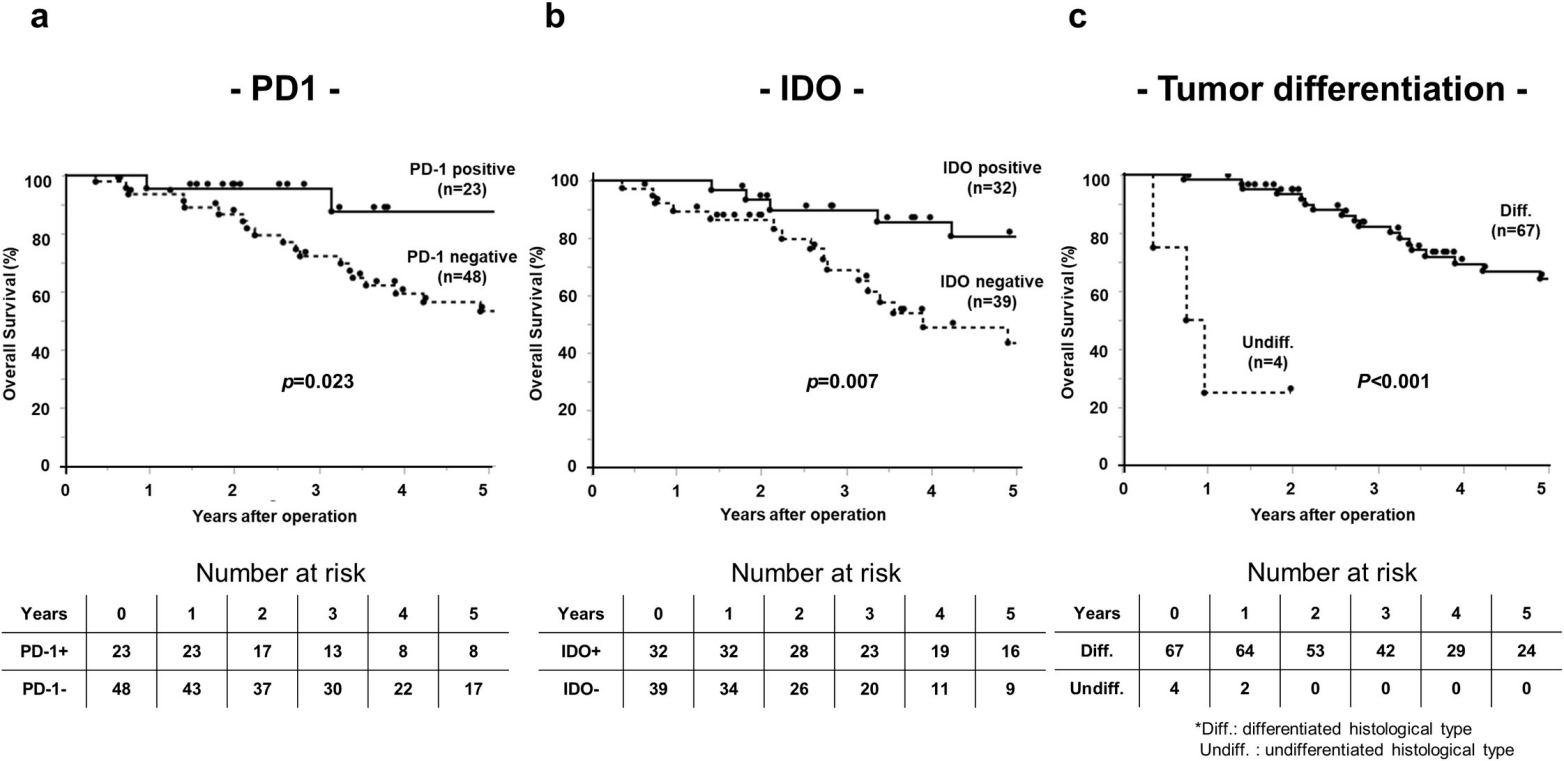
Overall survival rates of patients with CRLM post-hepatectomy according to PD-1, IDO expression and differentiation. a. PD-1 expression, b. IDO expression, c. Tumor differentiation.

Five-year OS was significantly better in the immune molecule-positive groups. The OS rate of the PD-1+ group was significantly better than that of the PD-1− group (87.7% vs 53.2%, *p =* 0.023) (Fig 3A), and the OS rate of the PD-L1+ group was significantly better than that of the PD-L1− group (82.4% vs 42.3%, *p =* 0.007). The OS rate of the IDO+ group was significantly better than that of the IDO− group (80.7% vs 43.5%, *p =* 0.007) (Fig 3B). The OS rate of the TAMs+ group was significantly better than that of the TAMs− group (82.6% vs. 48.0%, *p =* 0.005). The OS rate of the mTAMs+ group was significantly better than that of the mTAMs− group (79.9% vs 52.0%, *p =* 0.031). However, OS did not significantly correlate with cTAMs.

Univariate analysis of OS is shown in Table 5. H1 stage (*p =* 0.020), metastatic grade A (*p =* 0.007), low nomogram score (*p =* 0.020), differentiation in the primary site (*p*<0.001) (Fig 3C), shallow tumor invasion in the primary site (*p =* 0.040), immune molecule expression in CRLM (PD-1: *p =* 0.023, PD-L1: *p =* 0.007, IDO: *p =* 0.007), and TAMs and mTAMs in CRLM were identified as significant prognostic factors for better OS (TAMs: *p =* 0.005; mTAMs: *p =* 0.031). The apparent positive correlations between tumor size (*p =* 0.089) and metastasis period (*p =* 0.124) and OS were not statistically significant.

**Table 5:** Univariate and multivariate analysis of clinicopathological factors associated with overall survival post-hepatectomy.

Variables	5-year OS rate (%)	Univariate P-value	Multivariate analysis	
			HR (95% CI)	P-value
**< Metastatic tumor characteristics >**				
Age (<65 years / ≥65 years)	64.2 / 59.8	0.968		
Sex (men/women)	64.2 / 56.3	0.698		
Tumor maximum size (<5 cm / ≥5 cm)	65.3 / 50.0	0.089		
Tumor number (<4 cm / ≥4 cm)	66.4 / 52.8	0.269		
H-stage (H1/H2, 3)	72.7 / 44.4	0.020	0.36 (0.11–1.89)	0.291
Grade (A/B,C)	77.5 / 46.2	0.012	0.34 (0.08–1.39)	0.148
Metastasis period (synch/meta)	52.3 / 74.5	0.124		
Pre-operative chemotherapy (-: +)	60.2 / 100	0.307		
Post-operative chemotherapy (-: +)	71.1 / 59.0	0.586		
Nomogram preoperative score (<11 / ≥11)	81.8 / 49.3	0.020	0.50 (0.12–1.96)	0.292
PD-1 (-: +)	53.2 / 87.7	0.023	0.19 (0.03–1.10)	0.032
PD-L1 (-: +)	42.3 / 82.3	0.007	0.64 (0.23–1.77)	0.382
IDO (-: +)	43.5 / 80.7	0.007	0.37 (0.13–1.04)	0.049
TAMs (-: +)	48.0 / 82.6	0.005	0.66 (0.18–2.39)	0.520
cTAMs (-: +)	58.2 / 76.6	0.821		
mTAMs (-: +)	52.0 / 79.9	0.031	0.77 (0.21–2.85)	0.701
**< Primary tumor characteristics >**				
Colon / rectum	67.1 / 59.8	0.910		
Tumor differentiation (diff./ undiff.)	64.3 / 25.0	<0.001	0.02 (0.002–0.20)	*<*0.001
T (2,3/4)	74.4 / 35.2	0.040	0.62 (0.23–1.72)	0.470
Lymph node metastasis (-: +)	75.6 / 83.9	0.442		
Lymphatic invasion (-: +)	74.1 / 62.1	0.761		
Venous invasion (-: +)	78.5 / 58.3	0.515		

synch/ meta: synchronous/ metachronous, diff./undiff.: differentiated histological type/undifferentiated histological type.

Multivariate analysis revealed that PD-1 expression in CRLM (hazard ratio: 0.19, 95% CI 0.03–1.10, *p =* 0.032), IDO expression (hazard ratio: 0.37, 95% CI 0.13–1.04, *p =* 0.049), and differentiation (hazard ratio: 0.02, 95% CI 0.002–0.20, *p<*0.001) were independent prognostic indicators.

### Disease-free survival (DFS) according to immune molecule expression and TAMs

Univariate analysis of DFS is shown in Table 6. Metastatic grade A (*p =* 0.049), synchronous metastasis (*p =* 0.025), low nomogram score (*p =* 0.043), differentiation (*p =* 0.032), and IDO (*p =* 0.016) were identified as significant prognostic factors for better DFS. The DFS rate of the IDO+ group was significantly better than that of the IDO− group (41.1% vs 21.2%, *p =* 0.016). The DFS rate tended to correlate with PD-1(41.1% vs 26.5%, *p =* 0.175) and PD-L1 expression (37.5% vs 24.4%, *p =* 0.103), although the differences were not statistically significant. However, DFS did not correlate with high density of TAMs, mTAMs, and cTAMs.

**Table 6:** Univariate and multivariate analysis of clinicopathological factors associated with disease-free survival after hepatectomy.

Variables	5-year DFS rate (%)	Univariate P-value	Multivariate analysis	
			HR (95% CI)	P-value
**< Metastatic tumor characteristics >**				
Age (<65 years / ≥65 years)	21.8 / 37.0	0.715		
Sex (men/women)	35.3 / 22.0	0.770		
Tumor maximum size (<5 cm / ≥5 cm)	30.5 / 30.0	0.842		
Tumor number (<4 cm / ≥4 cm)	31.3 / 27.8	0.359		
H-stage (H1/H2, 3)	35.7 / 22.2	0.242		
Grade (A/B,C)	39.4 / 20.7	0.049	0.70	0.264
			(0.37–1.31)	
Metastasis period (synch/meta)	20.4 / 47.8	0.025	0.52 (0.24–1.13)	0.091
Pre-operative chemotherapy (-: +)	29.9 / 50.0	0.721		
Post-operative chemotherapy (-: +)	45.4 / 24.9	0.257		
Nomogram preoperative score (<11 / ≥11)	57.1 / 21.7	0.043	0.89	0.771
			(0.40–1.94)	
PD-1 (-: +)	26.5 / 41.1	0.175		
PD-L1 (-: +)	24.4 / 37.5	0.103		
IDO (-: +)	21.2 / 41.1	0.016	0.53 (0.29–0.99)	0.046
TAMs (-: +)	27.3 / 37.1	0.635		
cTAMs (-: +)	33.3 / 21.4	0.337		
mTAMs (-: +)	27.8 / 38.2	0.746		
**< Primary tumor characteristics >**				
Colon / rectum	31.3 / 30.3	0.668		
Tumor differentiation (diff./undiff.)	31.6 / 25.0	0.032	0.89 (0.41–1.94)	0.119
T (2,3/4)	33.7 / 21.4	0.454		
Lymph node metastasis (-: +)	39.6 / 23.0	0.061		
Lymphatic invasion (-: +)	33.4 / 29.9	0.668		
Venous invasion (-: +)	35.0 / 28.6	0.743		

synch/meta: synchronous/ metachronous, diff./undiff.: differentiated histological type/undifferentiated histological type.

Multivariate analysis revealed that IDO expression in CRLM (hazard ratio: 0.53, 95% CI 0.29–0.99, *p =* 0.046) was an independent prognostic indicator of DFS.

## Discussion

This study was conducted to investigate the prognostic significance of immune molecules in CRLM. To determine the clinical value of immune molecules, we measured PD-1, PD-L1, IDO and TAMs in CRLM simultaneously.

PD-1 and its ligand PD-L1 contribute to the regulation of the immune system and maintain peripheral tolerance through T-cell activation and attenuation [12]. PD-1 expression is upregulated in activated immune cells in response to virus infections and tumors. We previously found that PD-1/PD-L1 expression in primary stage II/III CRC tumors is associated with poor prognosis and correlates with the expression of TGF-β and forkhead box P3 (FOXP3) [10]. A meta-analysis found that PD-L1 expression in solid tumors, including CRC, is associated with shorter survival [23]. These studies showed that PD-L1 expression was related to poor tumor differentiation and could be an indicator of poor prognosis. However, PD-L1 expression in CRLM was significantly associated with well tumor differentiation in the present study. PD-L1 expression was also associated with lower frequency of lymph node metastasis. These less aggressive tumor characteristics which correlate with PD-L1 expression may contribute to better prognosis in CRLM. The mechanism of this association should be investigated in future study, but one possible explanation may be that PD-1/PD-L1 expression not only indicates activation of an immunoescape pathway but reflects the adaptive antitumor response to tumor antigens in the metastatic tumors.

The presence of TILs in liver tumors indicates a host immune response against disease progression. Previous studies found that the OS rate among patients with CD8+ CRLM is significantly higher than those with CD8− CRLM [24, 25]. Moreover, PD-L1 expression is associated with a high frequency of CD8+ TILs, suggesting that PD-L1 expression in CRLM signifies adaptive antitumor immunity, in which TILs are activated in response to tumor antigens. Another study found that a high CD8+/CD4+ ratio and a low FOXP3/CD8 ratio correlate with prolonged survival of PD-L1+ patients but not for that of PD-L1− patients with esophageal cancer [26]. This indicates that immune-induced and intrinsic oncogenic activation regulates PD-L1 expression.

TILs subsets including CD4, CD8, and FOXP3 should be evaluated, and the balance of tumor suppressive activity conferred by CD8+ Treg cells and CD4+ Treg cells and adaptive tumor responses should be determined in future experiments. Other possible explanations of conflicting results associated with PD-1/PD-L1 expression may be confounded by the use of different primary antibodies, inconsistent cut-off values, and microenvironmental crosstalk [27]. However, we used the same PD-1 and PD-L1 primary antibodies and the same staining evaluation methods as a previous study [10]. Our present results indicate that the prognostic significance of PD-1/PD-L1 expression in CRLM significantly differs from that of stage II/III CRC. Furthermore, PD-L1 expression in the primary tumor significantly correlated with that in CRLM. These findings suggest the possibility that PD-L1 expression in progressing tumors that generate metastasis possess features different from PD-L1 expression in primary tumors that have not yet generated metastasis.

This is the first report, to our knowledge, to show the prognostic significance of IDO expression in CRLM. IDO is expressed by tumor cells and tumor-draining lymph nodes; IDO arrests growth, induces cytotoxic T cells or natural killer cells to undergo apoptosis [28], induces host Tregs [29], and correlates with worse patient outcomes [30]. We previously found that IDO expression in stage III gastric cancer is associated with poor prognosis and immunotolerance through attenuating the activation of Tregs [30]. However, our present data show that IDO expression in CRLM correlated with more differentiated tumors and better prognosis associated with OS and DFS. Furthermore, IDO served as an independent prognostic factor for OS and DFS.

A recent report found a favorable prognostic role of IDO in locally advanced rectal cancer after neoadjuvant therapy [31]. Interestingly, tumors with high IDO expression possess high densities of infiltrating cytotoxic T lymphocytes (CD8+). These findings suggest IDO expression associated with immune activation reflects a local inflammatory milieu rather than immune tolerance. Intratumoral CD8+ lymphocytes produce IFN-γ, and IDO is secondarily induced in response to IFN-γ [32]. The local interaction between IDO and lymphocyte infiltration of metastatic sites may represent one mechanism that confers a favorable prognostic value upon IDO.

M2 macrophages express anti-inflammatory cytokines, increase angiogenesis, and promote tumor progression [33]. Recent studies show that M2-polarized macrophages as TAMs mainly express CD163, CD204, and CD206 [34, 35]. Another recent study reveals different roles for each subset of TAMs, resulting in different contributions to clinical outcomes [36]. CD163 is widely used to evaluate TAMs [21], and we used CD163 here as a marker for TAMs. Future studies should investigate the prognostic roles of different subsets of TAMs subsets in CRLM.

We previously reported that cell migration and the epithelial–mesenchymal transition are stimulated by TAMs in hepatocellular carcinoma [14]. However, the roles of macrophages and TAMs in CRC are controversial. Although some studies show that CRC macrophages possess anti-tumor activity and are associated with longer DFS [37], other studies found that macrophages correlate with tumor growth [38]. The present study shows that a high density of TAMs in CRLM correlates with good prognosis, possibly because of the localization of TAMs. mTAMs, which reside along the tumor margin and induce cancer cells to undergo apoptosis in a Fas ligand-dependent manner [39]. This anti-tumor effect of mTAMs is correlates with favorable OS of CRC [40]. Patients with CRC with high-density mTAMs experience lower hepatic metastasis rates and improved prognosis. It is predictable therefore that the marginal area of a tumor is required for the tumor-host interaction and the development of an antitumor immune response [41]. Consistent with these reports, we show here that high-density mTAMs were associated with good prognosis and lower tumor aggressiveness such as smaller tumors, lower H-stage, and lower grade.

Together, these data suggest that PD-L1, PD-1, IDO, and TAMs may act as an adaptive anti-tumor immunity rather than immune tolerance in CRLM. Notably, we found that PD-1, PD-L1, IDO, TAMs, and mTAMs were significantly associated with better 5-year OS of patients with CRLM in the univariate analysis. Moreover, we found that PD-1 and IDO in CRLM were independent prognostic markers for OS addition to the higher tumor differentiation. Among PD-1, PD-L1, IDO and TAMs, only IDO showed prognostic significance for 5-year DFS in the univariate analysis. Furthermore, IDO was the only one independent prognostic factor for DFS. In summary, only IDO was an independent prognostic factor for both OS and DFS, suggesting that IDO correlate with a more beneficial immune response and have a strongest impact for prognosis in CRLM.

This study had some limitations. First, this was a retrospective study conducted at a single institution study, with inherent risk for selection bias. Furthermore, this study only used one experimental method (immunohistochemistry) to evaluate protein expression in CRLM. Thus, expression should be confirmed at the mRNA level through a prospective study. Future research should analyze the association between TILs and immune molecules. Investigations of other subsets of TAMs will help reveal their additional roles in the tumor environment. Our study identified the expression profiles of different immune molecules in CRLM, and the mechanisms that regulate expression warrant further investigation.

## Conclusions

Our study demonstrates the importance of PD-1, PD-L1, IDO, and TAMs in influencing the clinical outcomes of patients with CRLM. These factors correlated with better OS and less aggressive features of CRLM. PD-1 and IDO were independent prognostic markers for OS. IDO showed prognostic relevance for DFS. Although further evaluation is required, these findings provide a compelling argument for using these molecules as markers to predict the prognoses of patients with CRLM.
